# PGPR Improves Barley Performance Under Saline Irrigation: Agronomic, Biochemical, and Transcriptional Evidence from a Two-Season Field Study

**DOI:** 10.3390/plants15121903

**Published:** 2026-06-19

**Authors:** Wessam A. Abdelrady, Jiasheng Xu, Li Hao, Yuqi Li, Elsayed E. Elshawy, Ashgan M. Abdel-Azeem, Sally E. El-Wakeel, Heba H. M. Alagamy, El-Shimaa E. I. Mostfa, Alaa El-Dein Omara, Nevein L. Eryan, Aziza A. Aboulila, Chenchen Zhao, Fanrong Zeng

**Affiliations:** 1 MARA Key Laboratory of Sustainable Crop Production in the Middle Reaches of the Yangtze River (Co-Construction by Ministry and Province), College of Agriculture, Yangtze University, Jingzhou 434023, China; wessam@yangtzeu.edu.cn (W.A.A.); 13736636202@163.com (J.X.); haoli.st@yangtzeu.edu.cn (L.H.); 2024720917@yangtzeu.edu.cn (Y.L.); 2 Department of Crop Science, Faculty of Agriculture, Qena University, Qena 83523, Egypt; 3 Barley Research Department, Field Crops Research Institute, Agricultural Research Center, 9 El-Gamaa Street, Giza 12619, Egypt; elsayed.elshawy@arc.sci.eg (E.E.E.); ashganabdelazeem2020@gmail.com (A.M.A.-A.); sallyelmorsy1@gmail.com (S.E.E.-W.); 4 Seed Technology Research Department, Field Crops Research Institute, Agricultural Research Center, 9 El-Gamaa Street, Giza 12619, Egypt; hebaalagamy2@gmail.com (H.H.M.A.); drshimaa3116@gmail.com (E.-S.E.I.M.); 5 Department of Microbiology, Soils, Water, and Environment Research Institute, Agricultural Research Center, 9 El-Gamaa Street, Giza 12619, Egypt; alaa.ahmed@arc.sci.eg; 6 Crop Physiological Research Department, Field Crops Research Institute, Agricultural Research Center, 9 El-Gamaa Street, Giza 12619, Egypt; nevingerges103@gmail.com; 7 Genetics Department, Faculty of Agriculture, Kafrelsheikh University, Kafr El-Sheikh 33516, Egypt; 8 Tasmanian Institute of Agriculture, University of Tasmania, Prospect, Launceston, TAS 7250, Australia; chenchen.zhao@utas.edu.au

**Keywords:** saline irrigation, *Azospirillum lipoferum*, plant growth-promoting rhizobacteria, proline, antioxidant enzymes, gene expression

## Abstract

Saline irrigation is a major constraint to crop production in newly reclaimed desert lands, even when pre-sowing soil salinity is low. This two-season field study evaluated whether plant growth-promoting rhizobacteria could improve barley performance under saline irrigation water with an electrical conductivity of 11.8 dS m^−1^ in the El Moghra region, Egypt. The barley cultivar Giza 2000 was grown under five inoculation treatments: an uninoculated saline-irrigated control; a single inoculation with *Azospirillum lipoferum*; and combined inoculations with *A. lipoferum* and *Bacillus coagulans*, *Bacillus circulans*, or *Enterobacter cloacae*. Because freshwater was unavailable at the experimental site, treatment effects were evaluated relative to the saline-irrigated control. Across both growing seasons, single inoculation with *A. lipoferum* produced the most consistent improvements in growth, yield formation, nutrient accumulation, soil biological activity, and seed nutritional quality. The combined treatment of *A. lipoferum* and *B. circulans* was generally the second-most effective. Bacterial inoculation also improved adjustment to physiological stress, as indicated by greater proline accumulation, lower antioxidant enzyme activities, and enhanced expression of stress-related genes associated with proline biosynthesis and secondary metabolism. Overall, the results indicate that *A. lipoferum* applied alone was more effective than the tested combinations of bacteria under saline irrigation. These findings provide field-based evidence that inoculant performance depends on strain composition and that single-strain inoculation can be a promising strategy for improving barley production in reclaimed sandy soils irrigated with saline water.

## 1. Introduction

Newly reclaimed land in arid and semi-arid regions is often irrigated with brackish or saline groundwater because freshwater resources are limited. In such systems, crop performance is governed by the combined effects of irrigation-water salinity, sandy soil texture, evaporative demand, and genotype rather than by salinity alone [1,2]. This distinction is important in reclaimed sandy soils, where low organic matter content, poor nutrient retention, and limited biological buffering can intensify the stress associated with saline irrigation [1]. Barley (*Hordeum vulgare* L.) is regarded as one of the more salt-tolerant cereal crops and is therefore a logical candidate for such environments. However, its productivity under saline irrigation still depends on maintaining osmotic adjustment, ion homeostasis, and control of oxidative stress [2,3,4].

The adverse effects of salinity on barley arise through both osmotic and ionic components of stress. The first response is the reduction in soil water potential, which restricts water uptake and slows early growth. At the same time, prolonged exposure leads to ion imbalance, membrane injury, metabolic disruption, and impaired grain formation [2,5]. In barley, tolerance is closely related to the capacity to regulate Na^+^/K^+^ balance, protect cellular structures from oxidative damage, and sustain stress-responsive metabolic pathways under field conditions [3,4]. For that reason, the relative salt tolerance of barley should not be interpreted as immunity to saline irrigation; rather, it indicates that this crop remains responsive to management practices that strengthen those protective processes [2,4].

Among such practices, plant growth-promoting rhizobacteria (PGPR) have received substantial attention because they can improve plant performance under salinity through several complementary mechanisms. These include biological nitrogen fixation, improved nutrient mobilization, stimulation of root growth, phytohormone production, enhancement of rhizosphere activity, and modulation of osmolyte accumulation [1,6]. These functions are particularly relevant in reclaimed sandy soils, where crop performance is strongly dependent on root–soil interactions and where microbial support may partially compensate for poor fertility and weak biological activity [1]. In cereals, *Azospirillum* and *Bacillus* are among the most studied PGPR groups because they combine rhizosphere competence with traits directly linked to plant nutrition and stress tolerance [6].

Evidence for the beneficial role of these bacteria in barley is already available. Earlier work showed that inoculation with salt-tolerant *Azospirillum* strains can improve barley growth under salinity conditions, including in the Giza 2000 cultivar [7]. More recently, the application of bacteria in saline-water irrigation has been reported to improve barley growth, nutrient uptake, antioxidant capacity, and productivity, confirming that microbial inputs can enhance field performance in saline environments [8]. Therefore, the present study is not intended to report the discovery of a novel biological mechanism. Its relevance lies more in practical application and specificity: to determine whether *Azospirillum lipoferum* performs more effectively as a single-strain inoculant or in combination with partner strains under the conditions of reclaimed sandy soils irrigated with saline water.

This issue is of great agronomic significance, as the superiority of microbial consortia cannot be presumed. Although strain co-inoculation may theoretically broaden functional versatility, field performance is governed by inter-strain compatibility, rhizospheric persistence, and the capacity of microbes to maintain metabolic activity under saline stress [1,6]. A two-season field comparison is therefore more informative than a simple assumption that multi-strain inoculation must outperform a single strain. In addition, evaluating treatment performance solely by yield can be insufficient under saline irrigation, because physiological adjustments also determine whether a treatment is truly beneficial. Traits such as proline accumulation, antioxidant enzyme activity, and the expression of stress-related genes provide useful complementary evidence for interpreting how inoculation influences barley adaptation under saline conditions [2,3,4]. Because PGPR-mediated salinity tolerance is commonly associated with improved nutrient acquisition, ion homeostasis, osmolyte accumulation, antioxidant regulation, and stress-related molecular responses [6,9,10,11], while the performance of microbial consortia depends on strain compatibility, rhizosphere establishment, and environmental context [12,13], we hypothesized that bacterial inoculation would improve barley growth, yield formation, nutrient status, physiological stress adjustment, and stress-related gene expression under saline irrigation. We further hypothesized that the magnitude of this improvement would depend on the inoculation strategy and that a well-adapted single strain, such as *A. lipoferum*, could perform as well as or better than the tested multi-strain combinations under reclaimed sandy-soil field conditions. Accordingly, this study compared single- and consortium-based bacterial inoculation treatments to improve barley performance under saline-water irrigation in reclaimed sandy soil.

## 2. Results

### 2.1. Environmental Context Under Saline Irrigation

The initial soil at El Moghra was extremely sandy and had low soluble salt levels before sowing (Table 1), whereas the irrigation water used throughout the experiment was highly saline (Table 2; EC = 11.8 dS m^−1^). Therefore, the present study should be interpreted as a field evaluation of barley grown in reclaimed sandy soil under saline irrigation water, rather than as barley grown in an inherently saline soil. This distinction is important for interpreting treatment effects because the experiment does not compare saline and non-saline environments; instead, it compares bacterial inoculation treatments under the same saline irrigation regime.

The soil data confirmed the coarse texture of the experimental site in both seasons, with total sand reaching 99.65% in 2022/2023 and 99.60% in 2023/2024, while the silt + clay fraction remained very low (0.35% and 0.40%, respectively) (Table 1). Soil pH was moderately alkaline, reaching 8.6 in the first season and 8.3 in the second, whereas the initial soil electrical conductivity (EC) remained low (0.10 and 0.13 dS m^−1^, respectively). Calcium carbonate content was also low, amounting to 1.30% in 2022/2023 and 0.90% in 2023/2024 (Table 1). These values indicate that the field soil itself was not strongly saline at sowing and that salinity stress during crop growth was mainly associated with the irrigation water source rather than with initial soil salinity. In contrast, the irrigation water applied uniformly to all treatments had a high salinity level, with EC = 11.8 dS m^−1^ and total dissolved solids (TDS) = 6731 mg L^−1^ (Table 1). The water was characterized by high concentrations of Na^+^ (1500 mg L^−1^) and Cl^−^ (3100 mg L^−1^), together with substantial levels of Ca^2+^ (348.8 mg L^−1^), Mg^2+^ (353.81 mg L^−1^), and SO_4_^2−^ (1284.9 mg L^−1^) (Table 2). Because all plots received the same irrigation water, differences observed among treatments should be attributed to bacterial inoculation rather than to variation in water quality.

Weather conditions during the active barley-growing period are summarized in Table 3. Mean air temperature ranged from 13.83 to 21 °C during the 2022/2023 season and from 14.31 to 22.32 °C during the 2023/2024 season. Reference evapotranspiration (ET_0_) increased progressively from winter to spring in both seasons, rising from 2.10 to 6.16 mm d^−1^ in 2022/2023 and from 2.14 to 6.94 mm d^−1^ in 2023/2024. This seasonal increase indicates that evaporative demand became greater toward the later stages of crop development, particularly during stem elongation and grain filling. Taken together, these environmental data show that the experiment was conducted in a combination of coarse-textured reclaimed soil, highly saline irrigation water, and increasing evaporative demand toward spring, providing the background against which the barley response to bacterial inoculation should be interpreted.

### 2.2. Plant Growth and Yield Formation of Barley Plants Under Saline Condition

**Figure 1:**
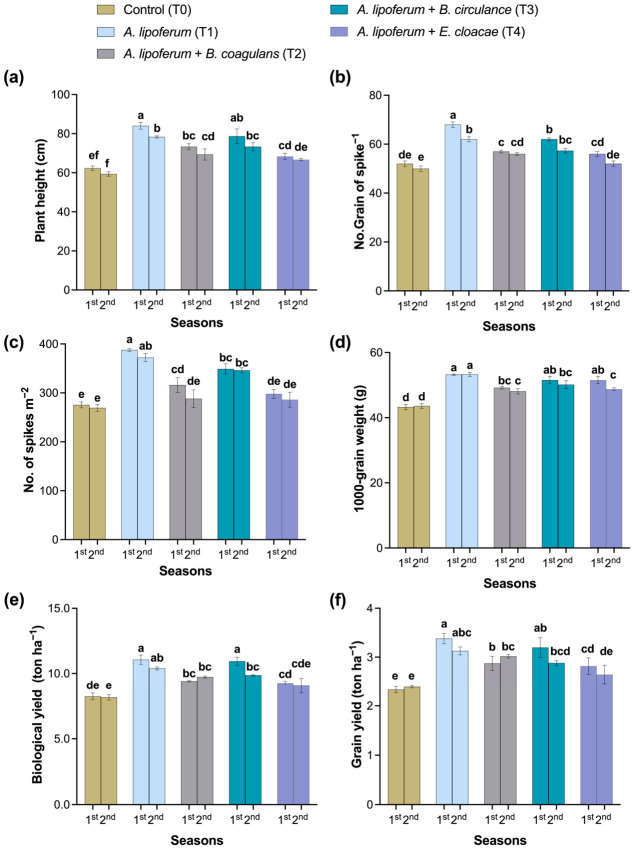
Growth and yield responses of barley to bacterial inoculation under bacterial inoculation and saline irrigation. (**a**) Plant height (cm), (**b**) no. of grains spike (grains spike^−1^), (**c**) no. of spikes (spikes m^−2^), (**d**) 1000 grain weight (g), (**e**) biological yield (ton ha^−1^) and (**f**) grain yield (ton ha^−1^). Treatments: T0, uninoculated saline-irrigated control; T1, *Azospirillum lipoferum* SP2; T2, *A. lipoferum* SP2 + *Bacillus coagulans*; T3, *A. lipoferum* SP2 + *Bacillus circulans*; T4, *A. lipoferum* SP2 + *Enterobacter cloacae*. Data are mean ± SD. Different letters indicate significant difference among the means at the *p* < 0.05 level.

Under the present experimental conditions, barley was grown in reclaimed sandy soil under saline irrigation; therefore, treatment effects should be interpreted as differences in performance between inoculated and uninoculated plants exposed to the same saline-water regime. Across both seasons, bacterial inoculation improved all measured growth and yield traits relative to the uninoculated control. However, the magnitude of the response differed among treatments (Figure 1). In general, T1 (*Azospirillum lipoferum*) produced the strongest response, whereas T3 (*A. lipoferum* + *Bacillus circulans*) was usually the second-most effective treatment.

Plant height increased with bacterial inoculation in both seasons (Figure 1a). The greatest increase was observed for T1, exceeding the control by 34.77% in the first season and 32.6% in the second season. T3 also markedly improved plant height, with increases of 25.67% and 23.6% in the two seasons, respectively, whereas T2 (*A. lipoferum* + *Bacillus coagulans*) and T4 (*A. lipoferum* + *Enterobacter cloacae*) produced smaller gains. A similar trend was observed for the number of grains per spike (Figure 1b). T1 increased this trait by 30.77% and 24% in the first and second seasons, respectively, while T3 increased it by 19.23% and 14.66%. The other treatments showed positive but smaller responses.

The number of spikes m^−2^ also increased under bacterial inoculation (Figure 1c). Again, T1 showed the strongest effect, with increases of 40.83% in the first season and 29.58% in the second season compared with the control (T0). T3 ranked second, whereas T2 and T4 produced lower improvements. The increase in spike density was accompanied by a corresponding improvement in 1000-grain weight (Figure 1d). T1 increased 1000-grain weight by 22.95% in the first season and 18.19% in the second season, while T2 and T3 showed intermediate responses, and T4 had the smallest effect.

The same pattern was observed in biological and grain yields (Figure 1e,f). T1 increased biological yield by 34.1% and 34.21% over the control in the first and second seasons, respectively, whereas T3 produced slightly smaller but still substantial increases. Grain yield showed the clearest treatment effect, with T1 exceeding the control by 44.9% in the first season and 31% in the second season (Figure 1f). The remaining inoculation treatments also increased grain yield, but to a lesser extent, and T4 consistently showed the weakest response. Overall, the agronomic data indicate that single inoculation with *A. lipoferum* was more effective than the tested mixed inoculations under saline irrigation.

### 2.3. Leaf Nutrient Status and Soil Dehydrogenase Activity of Barley Plants Under Saline Condition

**Figure 2:**
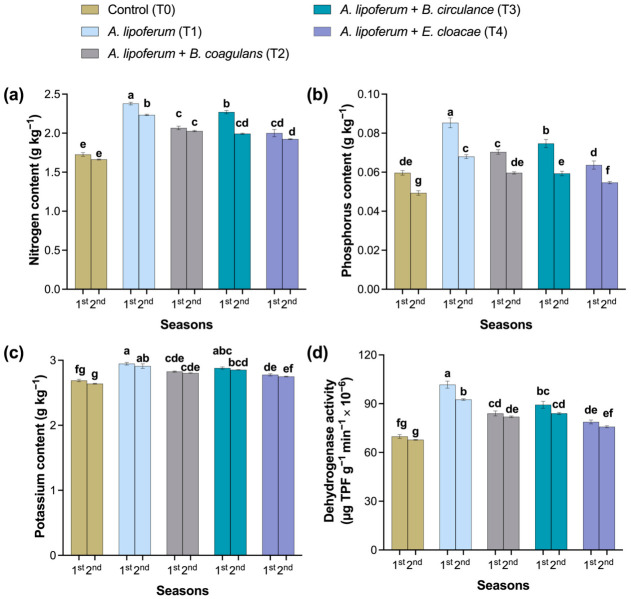
Leaf nutrient status and soil dehydrogenase activity of barley under bacterial inoculation and saline irrigation. (**a**) leaf nitrogen content (g kg^−1^), (**b**) leaf phosphorus content (g kg^−1^), (**c**) leaf potassium content (g kg^−1^), and (**d**) Dehydrogenase activity (µg TPF g^−1^ min^−1^ × 10^−6^). Treatments: T0, uninoculated saline-irrigated control; T1, *A. lipoferum* SP2; T2, *A. lipoferum* SP2 + *B. coagulans*; T3, *A. lipoferum* SP2 + *B. circulans*; T4, *A. lipoferum* SP2 + *E. cloacae.* TPF, triphenyl formazan. Data are mean ± SD. Different letters indicated significant difference among the means at the *p* < 0.05 level.

Leaf nutrient status and soil dehydrogenase activity were also enhanced by bacterial inoculation relative to the control (Figure 2). The clearest response was observed in leaf nitrogen content, where T1 increased N concentration by 37.57% in the first season and 34.34% in the second season (Figure 2a). T3 again ranked second, with increases of 31.21% and 19.88%, whereas T2 and T4 showed smaller improvements ranging from approximately 15.61% to 22.29% across the two seasons.

Phosphorus content followed a similar pattern (Figure 2b). T1 caused the largest increase, reaching 50% above the control in the first season and 40% in the second season. T2 and T3 showed moderate but clear increases, generally ranging from 16.67% to 20%, whereas T4 had no significant effect on leaf P in either season. In contrast, the increase in leaf K was more moderate across treatments (Figure 2c). T1 remained the best treatment, with increases of 9.67% and 10.23% in the first and second seasons, respectively, while the other treatments produced smaller but positive changes ranging from 3.35% to 7.95%.

Soil dehydrogenase activity increased across all inoculation treatments (Figure 2d), indicating enhanced biological activity in the rhizosphere. The highest values were recorded under T1, increasing dehydrogenase activity by 22.95% in the first season and 22.18% in the second season. The remaining treatments also improved dehydrogenase activity, with increases ranging from 10.25% to 19.09%. These findings show that bacterial inoculation not only improved plant nutrient status but also stimulated rhizosphere activity under saline irrigation conditions.

### 2.4. Grain Quality of Barley Under Saline Condition

**Figure 3:**
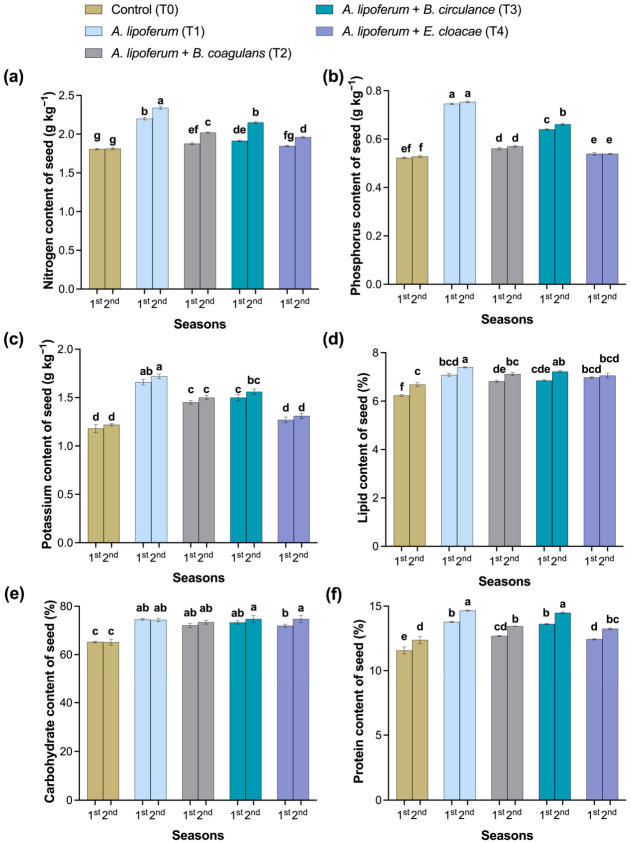
Seed nutritional quality of barley under bacterial inoculation and saline irrigation. (**a**) Nitrogen content (g kg^−1^), (**b**) phosphorus content (g kg^−1^), (**c**) potassium content (g kg^−1^), (**d**) lipid content (%), (**e**) carbohydrates content (%), and (**f**) protein content (%) in the seeds. Treatments: T0, uninoculated saline-irrigated control; T1, *A. lipoferum* SP2; T2, *A. lipoferum* SP2 + *B. coagulans*; T3, *A. lipoferum* SP2 + *B. circulans*; T4, *A. lipoferum* SP2 + *E. cloacae*. Data are mean ± SD. Different letters indicate significant difference among the means at the *p* < 0.05 level.

Bacterial inoculation improved seed nutritional composition and quality traits in both seasons (Figure 3). For seed nitrogen content, T1 again produced the largest increase, reaching 29.28% above the control in the first season and 22.91% in the second season (Figure 3a). T3 showed the next strongest response, whereas T2 and T4 produced smaller increases. Seed phosphorus content responded particularly strongly to T1, with increases of 47.06% in the first season and 50% in the second season compared with the control (Figure 3b). T3 also substantially improved seed P, while T2 and T4 had more limited effects.

A similar trend was observed for seed potassium content (Figure 3c). T1 increased seed K by 40.98% in the first season and 40.68% in the second season, followed by T3, T2, and then T4. Thus, T1 consistently gave the greatest improvement in seed macronutrient accumulation, and T3 generally ranked second. The same ranking was reflected in grain-quality traits. Lipid content increased under all inoculation treatments, with T1 recording increases of 13.64% in the first season and 10.78% in the second season (Figure 3d). T2 and T3 produced moderate increases, whereas T4 showed a smaller effect in the second season despite a relatively higher increase in the first season. Carbohydrate content also increased in response to bacterial inoculation (Figure 3e). T1 enhanced carbohydrate content by 14.31% and 14.09% in the first and second seasons, respectively, while T2, T3, and T4 also improved this trait, although to varying extents. Crude protein followed the same general pattern (Figure 3f), with T1 producing the largest increase (19.20% and 18.51% in the two seasons), followed closely by T3. Collectively, these results indicate that bacterial inoculation, especially with *A. lipoferum* alone, improved both grain yield and grain nutritional quality under saline irrigation.

### 2.5. Proline Accumulation and Antioxidant Enzyme Activities in Leaves of Barley Under Saline Conditions

**Figure 4:**
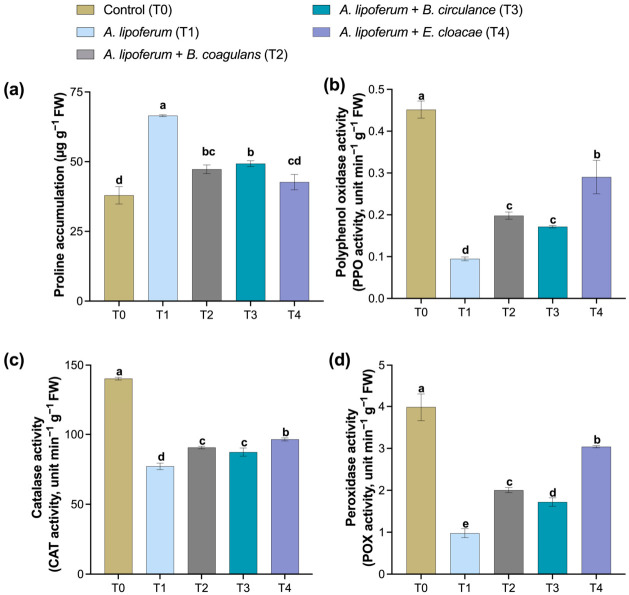
Proline accumulation and antioxidant enzyme activities in barley leaves under bacterial inoculation and saline irrigation. (**a**) Proline accumulation (µg g^−1^ FW), (**b**) polyphenol oxidase activity (PPO activity, unit min^−1^ g^−1^ FW), (**c**) catalase activity (CAT activity, unit min^−1^ g^−1^ FW), and (**d**) peroxidase activity (POX activit, unit min^−1^ g^−1^ FW). Treatments: T0, uninoculated saline-irrigated control; T1, *A. lipoferum* SP2; T2, *A. lipoferum* SP2 + *B. coagulans;* T3, *A. lipoferum* SP2 + *B. circulans;* T4, *A. lipoferum* SP2 + *E. cloacae*. Data are mean ± SD. Different letters indicate significant difference among the treatments at the *p* < 0.05 level.

Marked differences among treatments were also observed in proline accumulation and antioxidant enzyme activities (Figure 4). Proline content increased in all inoculated plants relative to the uninoculated control. Still, the greatest increase was observed in T1 (Figure 4a). In the control, proline concentration was 37.94 μg g^−1^ fresh weight (FW), whereas in T1 it increased to 66.50 μg g^−1^ FW, corresponding to a 75.28% increase. T3 and T2 also increased proline content to 49.24 and 47.30 μg g^−1^ FW, respectively, whereas T4 produced the smallest increase (42.70 μg g^−1^ FW, +12.55%). These results indicate that bacterial inoculation enhanced osmotic adjustment, with the strongest effect associated with T1. In contrast, the activities of polyphenol oxidase (PPO), catalase (CAT), and peroxidase (POX) were lower in inoculated plants than in the control (Figure 4b–d).

For PPO, the greatest reduction was observed under T1, where activity declined by 80% relative to the control (Figure 4b). T3 and T2 also reduced PPO activity by 62.22% and 55.56%, respectively, whereas T4 showed the smallest decrease (35.56%). Catalase activity followed the same pattern (Figure 4c). T1 reduced CAT activity by 45.01%, whereas T3, T2, and T4 reduced it by 37.74%, 35.39%, and 31.25%, respectively. For POX, the greatest reduction was observed in T1 (75.19%), followed by T3 (56.46%), T2 (49.37%), and T4 (23.04%) (Figure 4d). Taken together, these data suggest that inoculated plants, particularly those receiving T1, experienced lower oxidative stress than the uninoculated control under saline irrigation. Thus, the higher proline accumulation, together with lower activities of PPO, CAT, and POX, supports the view that bacterial inoculation improved physiological adjustment to salinity.

### 2.6. Gene Expression in Leaves of Barley Under Saline Condition

**Figure 5:**
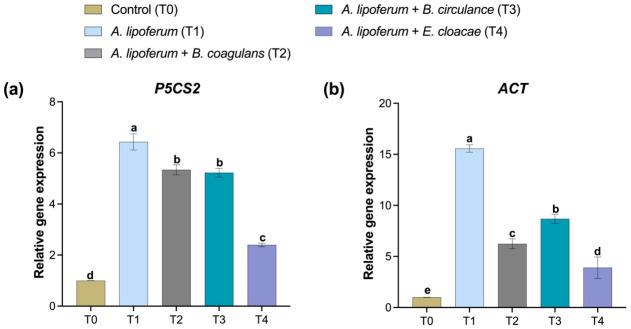
Expression of stress-related genes in barley leaves under bacterial inoculation and saline irrigation. (**a**) *P5CS2 (*gene for Δ^1^-pyrroline-5-carboxylate synthetase 2) and (**b**) *ACT* (gene for Agmatine coumaroyltransferase). Treatments: T0, uninoculated saline-irrigated control; T1, *A. lipoferum* SP2; T2, *A. lipoferum* SP2 + *B. coagulans*; T3, *A. lipoferum* SP2 + *B. circulans*; T4, *A. lipoferum* SP2 + *E. cloacae*. Data are mean ± SD. Different letters indicate significant difference among the treatments at the *p* < 0.05 level.

The qRT-PCR results further supported the physiological data by showing clear treatment-dependent differences in the expression of *P5CS2 *(gene for Δ^1^-pyrroline-5-carboxylate synthetase 2) (Figure 5a) and *ACT* (gene for Agmatine coumaroyltransferase) (Figure 5b). Transcript levels in the control were normalized to 1.0, and all inoculation treatments increased the expression of both genes relative to the control. The strongest induction was observed in T1, which increased *P5CS2* expression to 5.43-fold (Figure 5a) and *ACT* expression to 14.57-fold (Figure 5b) relative to the control. This strong transcriptional response is consistent with T1’s superior agronomic and physiological performance under saline irrigation.

The mixed inoculations also enhanced gene expression, although less strongly than T1. For *ACT* (Figure 5b), T3 increased *its* expression to 7.69-fold, followed by T2 (5.23-fold) and T4 (2.90-fold). For *P5CS2* (Figure 5b), T2 and T3 produced comparable responses, reaching 4.34-fold and 4.22-fold, respectively, whereas T4 showed the lowest induction (1.40-fold). Overall, the gene-expression analysis confirmed that bacterial inoculation activated stress-related pathways in barley grown under saline irrigation, with *A. lipoferum* alone showing the most pronounced effect.

## 3. Discussion

### 3.1. Superior Performance of Azospirillum lipoferum Single Inoculation over Combined Bacterial Consortia in Saline-Irrigated Barley

The present results should be interpreted as the response of barley to bacterial inoculation under saline irrigation conditions, where salinity acts through osmotic stress, ionic toxicity, nutrient imbalance, and oxidative damage to restrict growth and yield formation [2,9,10,14]. Within this framework, all inoculated treatments improved barley performance relative to the uninoculated control, but the response was consistently greatest with T1 (*A. lipoferum*) and generally second greatest with T3 (*A. lipoferum* + *B. circulans*). This pattern is agronomically important because the superiority of T1 was expressed across several yield-forming traits simultaneously, including plant height, spikes m^−2^, grains spike^−1^, 1000-grain weight, biological yield, and grain yield, indicating that inoculation improved whole-plant performance rather than a single isolated trait. This interpretation agrees with earlier barley studies showing that PGPR mitigate salinity-induced yield losses [7,15] and with more recent work showing that PGPR can improve crop performance under salinity by stabilizing plant water relations, ion homeostasis, and metabolic balance [9,10,11]. The superiority of *A. lipoferum* is also mechanistically plausible, because recent reviews of *Azospirillum* show that its effects are not explained by associative N fixation alone but by a combination of phytohormone production, stimulation of root branching, enhanced mineral acquisition, and activation of plant cellular responses that improve stress adjustment [16,17]. Under saline conditions, PGPR can further reduce stress ethylene through 1-aminocyclopropane-1-carboxylate (ACC) deaminase, improve Na^+^/K^+^ homeostasis, stimulate osmoprotectant accumulation, and enhance reactive oxygen species (ROS) detoxification, thereby improving both vegetative growth and reproductive success [9,10]. The fact that T1 outperformed the mixed inoculations should therefore be discussed directly, because recent work on multi-strain inoculants shows that consortia are not automatically superior; their success depends on compatibility, niche complementarity, biofilm behavior, and stable root colonization, whereas a single strain that is better adapted to the target environment may perform more consistently under field conditions [12,13].

### 3.2. PGPR Inoculation Modulates Soil Dehydrogenase Activity, Rhizosphere Function, and Plant Nutrient Acquisition Under Saline Irrigation

The increase in soil dehydrogenase activity (DHA) in inoculated treatments, especially under T1, indicates that bacterial inoculation improved rhizosphere biological activity under salinity rather than acting only as a superficial plant-growth stimulus. Because DHA is widely used as an indicator of oxidative metabolism in viable soil microorganisms, its reduction in the uninoculated saline control is consistent with salt-induced suppression of microbial respiration and organic-matter turnover. By contrast, the higher DHA values in inoculated plots suggest that the inoculants remained metabolically active in the rhizosphere and supported a more functional root zone. This interpretation is strengthened by recent evidence that microbial inoculants generally increase soil microbial biomass and restructure soil microbial communities. However, the magnitude of the response depends on environmental stress and soil nutrient status [18]. It is also consistent with recent PGPR studies showing that inoculation under salt stress can reorganize the rhizosphere microbiota, improve nutrient transformation, and enhance soil biological functioning, which together contribute to better crop performance [19,20]. Therefore, the higher DHA observed here is best discussed as a mechanistic bridge between inoculation and improved barley performance, because a more active rhizosphere is expected to strengthen nutrient turnover, root functioning, and stress buffering in saline sandy soils.

The higher N, P, and K concentrations in leaves of inoculated plants provide one of the clearest explanations for the yield response. In saline soils, nutrient acquisition is commonly impaired by reduced root growth, competition between Na^+^ and essential cations, and low nutrient-retention capacity in the rhizosphere. PGPR can counteract these constraints through several complementary mechanisms, including associative N fixation, phosphate solubilization, siderophore production, mobilization of mineral nutrients, stimulation of lateral root formation, and modification of rhizosphere chemistry [9,20]. Recent *Azospirillum*-focused reviews further show that this genus improves plant nutrition not only by supplying or mobilizing nutrients, but also by reshaping root architecture and enhancing the plant’s capacity to explore soil resources more efficiently [16,17]. The increase in K is especially significant under salinity conditions because maintaining a favorable K^+^/Na^+^ balance is central to salt tolerance and closely associated with improved physiological performance and yield stability [10,19]. Accordingly, the greater accumulation of N, P, and K in T1 and, to a lesser extent, T3 indicates that these treatments improved nutrient capture and ionic balance, which likely supported the better spike development, grain filling, and final yield observed in the present study.

### 3.3. PGPR Inoculation Enhances Salinity Adaptation via Modulating Proline Accumulation and Antioxidant Defense in Barley

The proline and antioxidant data indicate that bacterial inoculation improved physiological adjustment to salinity rather than merely increasing biomass. Proline is a major osmoprotectant in barley under salt stress, contributing to osmotic adjustment, stabilization of cellular structures, and limitation of oxidative injury, and recent work in barley has again confirmed its central role under salinity [21]. In the present study, the greater proline accumulation in inoculated plants, particularly under T1, should therefore be interpreted as part of a beneficial stress-adjustment response, as it occurred alongside improved nutrient status and higher yield. The antioxidant results point in the same direction. Salinity increases ROS formation, and PGPR-mediated salt tolerance is widely associated with more efficient regulation of antioxidant enzymes and lower oxidative damage [9,11]. Recent mechanistic studies further show that salt-tolerant rhizobacteria can improve plant performance by increasing K^+^/Na^+^ ratios, modulating abscisic acid (ABA)-linked stress responses, and reshaping the rhizosphere bacterial community, all of which reduce the physiological burden of salinity [19]. In addition, PGPR-derived exopolysaccharides can form root-adhering biofilms that bind Na^+^, retain moisture, and improve nutrient uptake, thereby reinforcing osmotic adjustment and antioxidant protection [9,10]. Thus, the lower oxidative damage and stronger biochemical adjustment observed in T1 and T3 are consistent with a more efficient stress-defense network in inoculated barley plants.

### 3.4. PGPR Inoculation Triggers Transcriptional Modulation of P5CS2 and ACT to Reprogram Proline Biosynthesis and Stress-Related Secondary Metabolism in Salinity-Stressed Barley

The transcriptional results strengthen the physiological interpretation of the study. *P5CS2* is directly associated with proline biosynthesis, and recent work continues to identify *P5CS* as a rate-limiting step in plant proline production under stress, including salinity [21,22]. Therefore, the stronger induction of *P5CS2* under T1 is fully consistent with the greater proline accumulation and superior agronomic performance of this treatment. The discussion of *ACT* should also be made more precise. *HvACT* encodes agmatine coumaroyltransferase, an enzyme involved in the biosynthesis of hydroxycinnamoylagmatines and hordatine-related secondary metabolites in barley, and recent work identified *HvACT-2HS1* as a functional gene in this pathway in barley seedlings [23]. Because these phenolic amide pathways are part of stress-responsive secondary metabolism, the stronger *ACT* expression in inoculated plants suggests that bacterial inoculation enhanced protective secondary-metabolism responses under salinity. Taken together, the upregulation of *P5CS2* and *ACT* under T1 indicates that *A. lipoferum* improved barley salt tolerance not only through rhizosphere-level effects on nutrient acquisition and soil biological activity but also through transcriptional reprogramming linked to osmoprotection and stress-related secondary metabolism. At the same time, these genes should be discussed as strong supportive markers of improved stress adjustment rather than as proof of a complete molecular mechanism, because the present experiment was designed to associate transcriptional changes with physiological and agronomic responses, not to resolve the entire signaling pathway.

## 4. Materials and Methods

### 4.1. Plant Material, Experimental Site, and Experimental Design

#### 4.1.1. Plant Material

Barley (*Hordeum vulgare* L.) cv. Giza 2000 was used as the test crop. Sowing was carried out on 1 December in both seasons. Seeds were hand-drilled at a rate of 143 kg ha^−1^, which is the recommended seeding rate for salt-affected soils under Egyptian conditions.

#### 4.1.2. Experimental Site

Field experiments were conducted at the Agricultural Research Center (ARC) in the El Moghra region, Egypt, a newly reclaimed desert area, during the 2022/2023 and 2023/2024 winter growing seasons. All plots were irrigated from the same groundwater source, and the quality of the irrigation water is presented in Table 2.

#### 4.1.3. Experimental Design

The experiment was arranged in a randomized complete block design (RCBD) with five treatments and three replicates. Each plot consisted of four rows, each 5.3 m long and 0.20 m apart, giving a total planted area of approximately 4.24 m^2^ per plot. The crop was grown on ridges under a drip-irrigation system.

### 4.2. Soil, Irrigation Water, and Crop Management

Before sowing each season, soil samples were randomly collected from the 0–30 cm layer of the experimental field for physical and chemical characterization. Composite soil samples were air-dried, gently crushed, passed through a 2 mm sieve, and stored in clean plastic containers until analysis. Soil texture was determined by particle-size analysis, and the percentages of total sand and silt + clay were calculated. Soil pH was measured in a 1:2.5 soil–water suspension using a calibrated pH meter, while soil electrical conductivity (EC) was determined in the soil extract using a calibrated EC meter. Calcium carbonate (CaCO_3_) content was determined following the standard procedures described by Page (1982) [24] and Cottenie et al. (1982) [25]. The soil properties are presented in Table 1.

Irrigation-water samples were collected from the groundwater source used for all plots and stored in clean polyethylene bottles until analysis. Water pH was measured electrometrically with a calibrated glass-electrode pH meter, and EC was determined at 25 °C with a calibrated electrical conductivity meter, expressed in dS m^−1^. Total dissolved solids (TDS) were determined from filtered water samples by the gravimetric residue method after drying at 180 °C. Soluble calcium (Ca^2+^) and magnesium (Mg^2+^) were determined by EDTA titration, while sodium (Na^+^) and potassium (K^+^) were determined using flame photometry. Carbonate (CO_3_^2−^) and bicarbonate (HCO_3_^−^) were determined by acid titration, chloride (Cl^−^) by argentometric titration with silver nitrate, and sulfate (SO_4_^2−^) by the barium chloride turbidimetric method. These analyses were performed following standard procedures for soil and irrigation water analysis [26,27] and standard water analysis methods [24,25,26,27]. The chemical composition of the irrigation water is shown in Table 2. Irrigation was applied through the drip-irrigation system from sowing until physiological maturity, approximately 110 days after sowing. About 55 irrigation events were applied during the growing season. Each irrigation event supplied approximately 60.6 m^3^ ha^−1^. Therefore, the total seasonal irrigation water applied was approximately 3333 m^3^ ha^−1^.

All plots received the same agronomic management throughout the two seasons. Irrigation, fertigation, weed control, and pest management were applied uniformly to all treatments. The fertilization program followed the ARC management schedule for the experimental site. It included acidification at establishment, phosphorus fertilization, split applications of nitrogen and potassium via fertigation, magnesium supplementation, and foliar micronutrient sprays. Thus, bacterial inoculation was the only experimental factor that differed among treatments.

### 4.3. Microorganisms and Inoculation Treatments

Four bacterial strains were used in this study: *Bacillus circulans* (NCAIM B.02324), *Bacillus coagulans* (NCAIM B.01123), *Azospirillum lipoferum* SP2, and *Enterobacter cloacae*. The strains were obtained from the Bacteriology Laboratory at the Sakha Agricultural Research Station, Kafr El-Sheikh, Egypt. *A. lipoferum* was cultured in semi-solid malate medium as described by Döbereiner and Day (1976) [28]. In contrast, the *Bacillus* strains and *E. cloacae* were grown in nutrient broth medium as described by Atlas (2004) [29]. Fresh bacterial cultures were incubated at 30 ± 2 °C for 48 h before inoculum preparation.

The field experiment included five bacterial inoculation treatments. The uninoculated treatment, T0, served as the saline-irrigated control and received no bacterial inoculation. Treatment T1 was a single-strain inoculation with *A. lipoferum* SP2. Treatments T2, T3, and T4 were combined inoculation treatments. Treatment T2 consisted of *A. lipoferum* SP2 + *Bacillus coagulans* (NCAIM B.01123), T3 consisted of *A. lipoferum* SP2 + *Bacillus circulans* (NCAIM B.02324), and T4 consisted of *A. lipoferum* SP2 + *Enterobacter cloacae*. *A. lipoferum* was cultured in semi-solid malate medium, whereas *B. coagulans*, *B. circulans*, and *E. cloacae* were cultured in nutrient broth. The *E. cloacae* strain was used as a local isolate and did not have an archived strain code. The bacterial treatment composition was also summarized in Table S1.

Bacterial concentrations were determined using the serial-dilution viable-count method. Briefly, 1 mL of each bacterial culture was serially diluted in 9 mL sterile distilled water or sterile saline solution, and appropriate dilutions were plated on the corresponding solid medium. Plates were incubated at 30 ± 2 °C for 48 h, and colonies were counted on plates containing 30–300 colonies. The bacterial population was expressed as colony-forming units per millilitre (CFU mL^−1^). Before use, each bacterial suspension was adjusted to approximately 1 × 10^8^ CFU mL^−1^.

The inocula were prepared as peat-based formulations. For single-strain treatments, 15 mL of the adjusted bacterial suspension was mixed aseptically with 30 g of sterilized peat carrier. For consortium treatments, the component strains were mixed in equal proportions immediately before addition to the sterilized carrier. The prepared inocula were kept in sterile sealed polyethylene bags under cool conditions until application and were protected from direct sunlight during handling. Seed inoculation was performed immediately before sowing at a rate of 1400 g ha^−1^. The inoculum was thoroughly mixed with barley seeds, and the treated seeds were left in the shade for approximately 20 min before sowing. Three additional applications of the corresponding bacterial preparations were delivered through the drip-irrigation system during the growing season.

### 4.4. Measured Traits and Analytical Procedures

Agronomic data were recorded from representative plants in each plot. Plant-level measurements were recorded from nine plants per treatment in each season, with three representative plants selected from each replicate plot. Plot means were then used for statistical analysis. The measured traits included plant height (cm), number of spikes (spikes m^−2^), number of grains per spike (grains spike^−1^), 1000-grain weight (g), biological yield (ton ha^−1^), and grain yield (ton ha^−1^). Leaf samples were collected at 60 days after sowing for macronutrient analysis. Nitrogen, phosphorus, and potassium contents were determined according to the method of Jones et al. (1991) [30]. Nitrogen was measured by the micro-Kjeldahl method following Peters et al. (2003) [31]; phosphorus, according to Page (1982) [24]; and potassium by flame photometry, according to Cottenie et al. (1982) [25].

For seed mineral analysis, dried seed samples were wet-digested according to the method of Peterburgski (1968) [32], and seed nitrogen, phosphorus, and potassium were determined as described by Mertens (2005) [33]. Total carbohydrates in barley seeds were estimated according to the method of Hedge et al. (1962) [34]. Lipid content was determined by Soxhlet extraction, and crude protein was calculated according to AOAC (2000) [35].

Soil biological activity was assessed by measuring dehydrogenase activity using the triphenyltetrazolium chloride (TTC) reduction method described by Casida Jr. et al. (1964) [36]. Leaf proline content was measured according to the method of Bates et al. (1973) [37]. Antioxidant enzyme activities were determined in fresh leaf extracts. Approximately 0.5 g of fresh barley leaf tissue was homogenized at 4 °C in 3 mL of 50 mM sodium phosphate buffer (pH 7.0) containing 50 mM Tris, 1 mM ethylenediaminetetraacetic acid disodium salt (EDTA-2Na), and 7.5% polyvinylpolypyrrolidone (PVPP). The homogenate was centrifuged at 12,000 rpm for 30 min at 4 °C, and the supernatant was used as an enzyme extract. Catalase (CAT) activity was assayed according to Aebi (1984) [38]. Peroxidase (POX) activity was determined according to Hammerschmidt et al. [39] by mixing 50 µL of enzyme extract with 2.9 mL of 100 mM sodium phosphate buffer (pH 6.0) containing 0.25% (*v*/*v*) guaiacol and 100 mM hydrogen peroxide (H_2_O_2_). The change in absorbance was recorded at 470 nm every 30 s for 3 min, and POX activity was expressed as the increase in absorbance min^−1^ g^−1^ fresh weight. Polyphenol oxidase (PPO) activity was determined according to Hammerschmidt et al. [39] by mixing 100 µL of enzyme extract with 1.5 mL of 0.1 M sodium phosphate buffer (pH 6.5), followed by adding 200 µL of 0.01 M catechol to initiate the reaction. The change in absorbance was recorded at 495 nm, and PPO activity was expressed as the increase in absorbance min^−1^ g^−1^ fresh weight.

### 4.5. RNA Extraction and Quantitative Real-Time PCR Analysis

Total RNA was isolated from 100 mg of barley leaf tissue using the easy-spin™ Total RNA Extraction Kit (Cat. No. 17221; iNtRON Biotechnology, Seongnam-si, Gyeonggi-do, South Korea) according to the manufacturer’s instructions. RNA quantity and purity were assessed spectrophotometrically, and RNA integrity was verified by electrophoresis on 2% agarose gel. First-strand cDNA was synthesized from 2 µg of total RNA using the TOPscript™ cDNA Synthesis Kit (Cat. No. EZ005S; Enzynomics Co. Ltd., Daejeon, South Korea). Quantitative real-time PCR (qRT-PCR) was performed in a final reaction volume of 20 µL containing 10 µL TOPreal™ qPCR 2× PreMIX (SYBR Green, low ROX; Enzynomics Co. Ltd., Daejeon, South Korea), 0.5 µM of each primer, and 2 µL of cDNA template. Amplification was carried out on a Bio-Rad C1000 thermocycler (Bio-Rad, Hercules, CA, USA). Relative transcript abundance of *ACT* ( gene name of agmatine coumaroyltransferase) and *P5CS2* (gene name of Δ^1^-pyrroline-5-carboxylate synthetase 2; proline biosynthesis) was determined using Hvα-tubulin as the internal reference gene. Relative expression levels were calculated according to the 2^−ΔΔCt^ method of Livak and Schmittgen (2001) [40]. The primer sequences used in the qRT-PCR assays are listed in Table S2.

### 4.6. Statistical Analysis

All measurements were performed across three biological replicates. Nine individual plants were sampled per treatment each season, with three randomly selected plants taken for each replicate. Prior to statistical analysis, data normality was tested using the Shapiro–Wilk test, and homogeneity of variances was tested using the Levene’s test. Only datasets satisfying both statistical assumptions at *p* > 0.05 were subjected to analysis of variance (ANOVA) appropriate for the randomized complete block design (RCBD) in IBM SPSS Statistics (version 29.0.1.0; Armonk, NY, USA). Significant differences among the means and treatments were compared using Tukey HSD post hoc test at *p* ≤ 0.05. Figures were generated in GraphPad Prism 10 (version 10.2.0; Boston, MA, USA).

## 5. Conclusions

In reclaimed sandy soil under saline irrigation with groundwater at 11.8 dS m^−1^, bacterial inoculation improved barley performance relative to the uninoculated control over two successive seasons. Among the tested treatments, *A. lipoferum* applied alone produced the most consistent and pronounced responses, resulting in the highest values for growth, yield, nutrient status, proline accumulation, soil dehydrogenase activity, and *ACT* and *P5CS2* expression. The *A. lipoferum* + *B. circulans* consortium was the second-most-effective treatment, whereas the other mixed inoculations did not surpass the single-strain inoculant. Thus, the proposed hypothesis was supported by the results: bacterial inoculation improved barley performance under saline irrigation, and the treatment’s effectiveness depended on the inoculation strategy. In particular, single inoculation with *A. lipoferum* produced the strongest improvements in growth, yield, nutrient status, proline accumulation, soil dehydrogenase activity, and *ACT* and *P5CS2* expression, confirming that a well-adapted single strain can outperform more complex bacterial consortia under the field conditions tested. These findings indicate that, under the conditions of this study, the agronomic value of PGPR lies not in the complexity of the formulation but in the effective selection of a well-adapted inoculant. Therefore, *A. lipoferum* can be considered the most promising treatment for improving barley performance under saline irrigation in newly reclaimed soils. Future research should include a freshwater control, monitoring root-zone salinity throughout the season, documenting irrigation volumes and frequency, and verifying the identity, dose, compatibility, and field persistence of the inoculant before making broader recommendations.

## Figures and Tables

**Table 1:** Soil properties before sowing at 0–30 cm depth for El Moghra during the 2022/2023 and 2023/2024 seasons.

Variable	2022/2023	2023/2024
Total sand (%)	99.65	99.60
Silt + clay (%)	0.35	0.40
Soil pH	8.6	8.3
Soil EC (dS m^−1^)	0.10	0.13
CaCO_3_ (%)	1.30	0.90

Values represent soil properties of the 0–30 cm soil layer before sowing in each season. EC, electrical conductivity; CaCO_3_, calcium carbonate.

**Table 2:** Chemical analysis of water irrigation.

Parameters	pH	EC (dS m^−1^)	TDS (mg L^−1^)	Cations (mg L^−1^)				Anions (mg L^−1^)			
				Ca^2+^	Mg^2+^	Na^+^	K^+^	CO_3_^2−^	HCO_3_^−^	SO_4_^2−^	Cl^−^
Results	6.3	11.8	6731	348.8	353.81	1500	29	0	230	1284.9	3100

Values represent the chemical composition of the groundwater source used uniformly for irrigation in all treatments. EC, electrical conductivity; TDS, total dissolved solids.

**Table 3:** Monthly weather summary for the active barley cropping period derived from the uploaded supplementary workbook.

Month	2022/2023 Air Temperature (°C)	2022/2023 ET_0_ (mm d^−1^)	2023/2024 Air Temperature (°C)	2023/2024 ET_0_ (mm d^−1^)
December	16.63	2.10	16.87	2.14
January	15.09	2.34	14.31	2.74
February	13.83	2.92	15.07	3.21
March	18.41	4.76	18.10	4.71
April	21.00	6.16	22.32	6.94

Air temperature values are monthly means of daily average air temperature. ET_0_ = reference evapotranspiration.

## Data Availability

The original contributions presented in this study are included in the article. Further inquiries can be directed to the corresponding authors.

## Supplementary Materials

The following supporting information can be downloaded at https://www.mdpi.com/article/10.3390/plants15121903/s1: Table S1: Bacterial inoculation treatments used in the field experiment; Table S2: Primers used for quantitative real-time PCR analysis.

## Abbreviations

The following abbreviations are used in this manuscript:

PGPR	Plant growth-promoting rhizobacteria
ARC	Agricultural Research Center
RCBD	Randomized complete block design
EC	Electrical conductivity
TDS	Total dissolved solids
TTC	Triphenyltetrazolium chloride
CAT	Catalase
POX	Peroxidase
PPO	Polyphenol oxidase
LSD	Least significant difference
ET_0_	Reference evapotranspiration
DHA	Dehydrogenase activity
ACC	Ethylene through 1-aminocyclopropane-1-carboxylate
ROS	Reactive oxygen species
CFUs	Colony-forming units
cDNA	Complementary DNA
TPF	Triphenyl formazan
qRT-PCR	Quantitative real-time PCR
*ACT*	Agmatine coumaroyltransferase
*P5CS2*	Δ^1^-pyrroline-5-carboxylate synthetase 2
EDTA-2Na	Ethylenediaminetetraacetic acid disodium salt
PVPP	Polyvinylpolypyrrolidone
H_2_O_2_	Hydrogen peroxide
