# Supplementary material for: PGPR Improves Barley Performance Under Saline Irrigation: Agronomic, Biochemical, and Transcriptional Evidence from a Two-Season Field Study
Source: Plants (Basel). 2026 Jun 19;15(12):1903. doi: 10.3390/plants15121903 (PMC13306323; doi:10.3390/plants15121903)
Supplement: Supplementary file 1 [file plants-15-01903-s001.zip › plants-4326174-supplementary.pdf]

Table S1. Bacterial inoculation treatments used in the field experiment.

Treatment	Microbial composition	Culture medium/note
T0	No bacterial inoculation	—
T1	*Azospirillum lipoferum* SP2	Semi-solid malate medium
T2	*A. lipoferum* + *Bacillus coagulans* (NCAIM B.01123)	Semi-solid malate + nutrient broth
T3	*A. lipoferum* + *Bacillus circulans* (NCAIM B.02324)	Semi-solid malate + nutrient broth
T4	*A. lipoferum* + *Enterobacter cloacae* isolate	Semi-solid malate + nutrient broth; strain code not archived

Table S2. Primers used for quantitative real-time PCR analysis.

No.	Primer name/gene putative function	Primer sequences (5' → 3')
1	*Act /Agmatine coumaroyl transferase*	Forward: CAATGTTCCTGCCATGTACG<br>Reverse: AGCGAGATCCAAACGAAGAA
2	*P5C2.2 / Proline*	Forward: GGACCTGTTGCACATGACAC<br>Reverse: TCACAGAACCTTGTGCTTGC
3	*HVα-tubulin / Reference gene*	Forward: AATGCTGTTGGAGGTGGAAC<br>Reverse: GAGTGGGTGGACAGGACACT
